# A Quarrel Ended

**Published:** 1871-10

**Authors:** 


### "A QUARREL ENDED.

For many months the profession in Georgia have been agitated by a bitter quarrel, in which the State Medical Society and the Atlanta Medical College were parties. We are pleased to learn from the *Atlanta Medical Journal* that the difficulties have all been settled, so as to secure "permanent fraternal relations," and that, in the words of the editor, "the medical profession of Georgia are now in peace, and are determined no longer to be a reproach to science and to humanity."

The above we clip from the *Pacific Medical and Surgical Journal.* Were it not for the respect we feel is due the gentlemanly and courteous editors of the above journal, and that silence on our part might be interpreted as an *endorsement* of "*a quarrel ended,*" we should not again notice a controversy we had hoped was, by this time, fully understood by the profession. In justice, however, to the profession of Georgia, as their representative, we cannot allow the above to go by uncorrected. In saying this we would not have it understood that we blame our brothers of the "Pacific Slope." On the contrary, we can appreciate the kindness of heart manifested in their rejoicing over a supposed adjustment of a professional "quarrel."

There is either *a principle*, or *no principle*, involved in the issues from which originated the medical controversy in Georgia:

or to change the terms, there are parties in this "quarrel", as in every other, who are maintaining the RIGHT, as opposed to the WRONG. If this be a true statement, then until the RIGHT is vindicated and the WRONG pronounced WRONG, there can be no genuine "harmony" between contending parties.

How does the case stand in Georgia? In order to answer this question, it will be necessary to revert, for a moment, to the causes which evoked the controversy (so-called) between the Atlanta Medical College and the Georgia Medical Association. In reviewing these, we shall speak "to the facts" candidly and truthfully. The writer, even were it legitimate (which he does not admit), cannot afford to forfeit his self-respect by misrepresentation and falsehood, in order to indulge in revenge (which he does not feel), or to carry a point (which is not worth the sacrifice) by resorting to such means. Sooner or later the history embodying the whole truth, in regard to the controversy referred to, will be fully known and understood, and the writer would "hang his head in shame" should the charge of falsehood, in so small a matter, be truthfully fastened upon him by his professional brethren. What we shall write, therefore, shall pertain to the *facts*, as we understand them, drawn from the records.

What gave origin to "the controversy" between the Atlanta Medical College and the Georgia Medical Association? Without going behind the difficulties between the Board of Trustees and the Faculty of that College, to invoke an hypothisis as to the *motives* which originated the strife between these, *facts* show that the *germ* of the quarrel sprung from the assumption on the part of the Faculty of said College of "powers" which the Georgia Medical Association declared were "unusual and extraordinary;" "powers," which had been, according to the statement of the Board of Trustees, "fraudulently and clandestinely obtained" by one of the Faculty while a member of the Legislature. With the quarrel between members of the Faculty on the one hand, and between a portion of the Faculty and the Board of Trustees on the other, the profession, in its organized capacity, had nothing to do—whatever may have been their sympathies. Not one fact can be adduced by which it can be logically demonstrated that the Association, in its dealings with this College question, had any other than the good of the profession at heart—which good was ultimately accomplished, in one particular at least, by the subsequent admissions made by the Faculty, *after* the repeal of the obnoxious amendment to the original charter, in which those "unusual and extraordinary powers" were incorportated. Every intelligently informed physician of this State is well aware that *long before* the "College question" reached the profession, in its Associational capacity, there was "*war*" between the Faculty and the Board of Trustees of that College, caused by the "amendment" to the original charter, which the Trustees openly declared had been procured "without their consent or knowledge." Among these parties this was a contest for supremacy in the administration of the College, involving a legal question, as to the rights of each under its charter—in which contest the Faculty assumed, by the amendment, "powers," which conferred the right upon them to seat and unseat any professor at pleasure, and at any time, and under any cirumstances, to graduate students without the consent or approval of the Board of Trustees. So fierce had the war grown that the Board rejected the amendment, repudiated the acts of the Faculty and refused to co-operate with them as organized under the powers of the amendment In despite of this, the Faculty proceeded to make all the appointments, and graduated students not only independenty of the Board, but against their protest.

The Trustees and Faculty were waging a war against each other as seen above, when the Georgia Medical Association was informed as to the *ethical character of the Amendment under which the Faculty was then operating.* The illegality of a "fraudulently procured" amendment was assailed by the Trustees, and upon this fact, viewing it in its *ethical bearings*, as both illegal and unprofessional, the Association passed the following preamble and resolution at its annual meeting, held in Augusta, in 1868:

"WHEREAS, The above amended charter confers unusual and extraordinary powers upon the Faculty of the Atlanta Medical College, whereby they are authorized to confer the degree of M. D. on persons, regardless of time or condition, save as to said Faculty may seem fit and proper; therefore,

*Resolved*, That we cannot recognize the graduates of said College that may hereafter receive their diplomas under the amended charter aforesaid."

Did this action of the Association deter the Faculty? Not in the least! They opened the doors of the College, and in defiance of the protest of the Trustees and the action of the Association, graduated 26 young men! Nay, more—they filed a bill of injunction against the Trustees, restraining them from vacating the chairs of the Institution!! Thus the "war" was begun, and thus it progressed. Before the next meeting of the Association the Trustees, upon the assembling of the Legislature, petitioned that body to repeal the "Amendment," which effort was met by the Faculty in a counter "memorial," in which they desired the Legislature to "sustain" the "unusual" and "extraordinary powers" contained in the Amendment, and, in order to show their contempt, both for the ethics and the organized profession of the State, they charged that the action of the Association, in reference to the Amendment, had in it "an utter absence of the truth," and that the "Annual Session of the Association, in 1868, was a *Meeting* of Physicians *assuming* to *represent* the Medical Profession of the State, and that such Annual Meeting was made up, almost entirely, of the Dr. Powell clique, of the city of Atlanta, and of members of rival Schools, and the whole affair (Association Meeting) was gotten up and consummated for the purpose of injuring the Atlanta Medical College; that the voice of the State Medical Society and of the Profession was not heard, etc., etc."

This "memorial" was printed in various newspapers, in pamphlet form, and distributed throughout the State.

Before the next meeting of the Association, a number of the Trustees having resigned, the remainder effected a compromise with the Faculty, by agreeing to add nine new members to the Board; the Faculty consenting to the repeal of the Amendment, and agreeing (upon an understood programme,) to a re-organization of the Faculty. To say that the old members of the Board were decieved by this strategy, they themselves, now admit; for it soon became apparent that, under a change of name, they still exercised, virtually, all the powers of the Amendment. In proof of which we will here give an unpublished *fact.* The new Board, under pretext of conformity to the stipulations of the compromise, advertised the chairs vacant, and requested written applications from the profession generally in order, by a fair election, as was supposed, to refill the chairs. Under the belief that these gentlemen *intended* to carry out what they had published, a gentleman of a neighboring city, made application for a certain chair. He was replied to by the former occupant of that chair, and was informed, to his astonishment, that "*it was understood*" that *there would be no changes among the old members of the Faculty—which was verified to the letter*, *upon the meeting of the Board.* How was that knowledge obtained? May there not be medical as well as political rings? This, however, was not known to the Association, which met in Savannah in 1869. At that meeting, the Association, by resolution, removed the charge of irregularity so far as the charter was involved, "provided the present Faculty, under the new charter, will repudiate the action of the Faculty existing at the time of the passage of the resolution, during the meeting at Augusta." It also required of the Faculty "a distinct and unequivocal withdrawal of the objectionable language used in the memorial, and that such withdrawal must be through the public journals of the State." At that meeting the old Faculty offered an apology, disavowing "any purpose to reflect upon the Medical Association of Georgia." This would seem upon its face to have been ample, but was no apology, nor was it intended as such. The Faculty apologized to a body against whom, they said, they had not sinned—the Georgia Medical Association. Their language could not apply to that body, since they had assailed another *assuming* to be the Association and not that body itself! The Association, therefore, rejected the "sham" apology and required "a distinct and unequivocal" one.

The new Board, with the Faculty, moved on in defiance of the action of the Association, administering the affairs of the College and graduating students.

Wishing, doubtless, to delude the profession rather than to show respect to the Association, the Board requested the *new* Faculty to send an additional apology to the next meeting of the Association. Thus urged, the Faculty, through the Trustees, forwarded another apology. Four of the members of the Board, when the document was read, objected to its being sent, declaring that an Association of honorable gentlemen could not receive it, being clearly an extensive dilution of the one offered at Savannah. But this opinion, as to its spirit, was not an assumption on the part of those four gentlemen. The representatives of the Faculty, when presenting the apology to the Trustees, were asked by them, "Is it understood that the Faculty agree to recognize the meeting held at Augusta, as a meeting of the Georgia Medical Association?"

To which one of the Faculty replied: "*We will never recognize the meeting at Augusta, as one of the Georgia Medical Association.*" Nevertheless, this apology was offered, *as an apology*, at the meeting of the Association, at Macon! One of the Trustees being a member of the body, protested against its being received. It was rejected, and the Faculty, after three years grace, were expelled.

Thus matters stood, when an ex-member of the Faculty, (who, it is said, was delighted with the action of the Association in expelling his former friends) after the meeting of the American Medical Association, in which certain principles were announced which precluded this "magnificent weather-cock" from running two opposing schedules, at the same time losing sight of the principles of the ethics, made a "dash" for the strong side, and "turned up" the President of the Atlanta Academy of Medicine, which Academy, we are informed, he declared should "control and regulate" the profession of this city. How this Academy originated and who constituted its membership, will be seen by referring to the reports of the Delegate of the Fulton County Medical Society and its Committee. By a careful reading, also, of these reports, our Pacific friends, will get a clear insight as to the ways and means employed by the College party in their late efforts to overthrow the recorded action of the State Association against themselves. We will see that the "history" of the controversy, as contained in the above mentioned pamphlet, shall be placed in the hands of our California friends. Should they carefully read it, they will be prepared to say, whether or not, "the difficulties have all been settled, so as to secure '*permanent fraternal relations*' and that 'the medical profession of Georgia *are now in peace.*'"

